# Regulation of Monocarboxylic Acid Transporter-1 by cAMP Dependent Vesicular Trafficking in Brain Microvascular Endothelial Cells

**DOI:** 10.1371/journal.pone.0085957

**Published:** 2014-01-16

**Authors:** Amy L. Uhernik, Lun Li, Nathan LaVoy, Micah J. Velasquez, Jeffrey P. Smith

**Affiliations:** Department of Biology, Colorado State University-Pueblo, Pueblo, Colorado, United States of America; University of Naples Federico II, Italy

## Abstract

In this study, a detailed characterization of Monocarboxylic Acid Transporter-1 (Mct1) in cytoplasmic vesicles of cultured rat brain microvascular endothelial cells shows them to be a diverse population of endosomes intrinsic to the regulation of the transporter by a brief 25 to 30 minute exposure to the membrane permeant cAMP analog, 8Br-cAMP. The vesicles are heterogeneous in size, mobility, internal pH, and co-localize with discreet markers of particular types of endosomes including early endosomes, clathrin coated vesicles, caveolar vesicles, trans-golgi, and lysosomes. The vesicular localization of Mct1 was not dependent on its N or C termini, however, the size and pH of Mct1 vesicles was increased by deletion of either terminus demonstrating a role for the termini in vesicular trafficking of Mct1. Using a novel BCECF-AM based assay developed in this study, 8Br-cAMP was shown to decrease the pH of Mct1 vesicles after 25 minutes. This result and method were confirmed in experiments with a ratiometric pH-sensitive EGFP-mCherry dual tagged Mct1 construct. Overall, the results indicate that cAMP signaling reduces the functionality of Mct1 in cerebrovascular endothelial cells by facilitating its entry into a highly dynamic vesicular trafficking pathway that appears to lead to the transporter's trafficking to autophagosomes and lysosomes.

## Introduction

Monocarboxylic acid transporter 1, Mct1, is a ubiquitous transmembrane protein that facilitates proton coupled symport of important cellular energy substrates such as lactate and other monocarboxylates across plasma membranes [1], [2]. This makes it essential for the normal energy metabolism of cells and gives it pathophysiological importance for diseases in which monocarboxylate metabolism is a component. While the basic mechanics of its transport function is well understood, limited progress has been made in understanding the acute cell-signaling dependent regulation of Mct1, however, elucidating such mechanisms will promote development of new treatments for diseases involving monocarboxylic acid transport. The focus of this work was to elucidate mechanisms of acute regulation of Mct1 function in brain capillary cells. In brain, Mct1 facilitates an intercellular transport of lactic acid from astrocytes to neurons which is required for learning and memory [3], and it has important roles in brain cancer that point to it as a therapeutic target [4], [5]. Both of these involve a significant microvascular component that would likely involve acute cell signaling dependent Mct1 regulation, but this has not been well investigated in brain. In the blood-brain barrier, Mct1 is the only mechanism to transport lactic acid from brain to blood giving it a role in brain diseases such as stroke and injury where the level and time-course of cerebral lactic acidosis is a key etiological component [6], [7], [8]. Mct1 has also been targeted in cerebrovascular endothelial cells as a potential facilitator of blood to brain drug delivery [9], [10]. Therefore, it is important to understand basic mechanisms that regulate Mct1 function in cerebrovascular endothelial cells since they present specific targets for therapeutic drug development to treat brain diseases ranging from learning and memory disorders to stroke and cancer, and could enhance delivery of pharmaceuticals to brain.

It was previously shown that the β-adrenergic pathway regulates Mct1 function in the rat brain capillary endothelial cell line (RBE4) by reducing plasma membrane levels of the transporter in a mechanism involving its cAMP-dependent vesicular trafficking [6], [8]. Microscopic analysis first showed a punctate immunostaining pattern for Mct1 in the cytoplasm of RBE4 cells suggesting its localization to cytoplasmic vesicles [7], while later work showed Mct1 puncta colocalizing with antibodies to clathrin, caveolin-1, EEA1, and Rab11, consistent with its presence in clathrin coated vesicles, caveolae, early endosomes, and recycling endosomes [8]. However, the immunostaining process can produce artifacts involving protein extraction and relocalization, making it necessary to compliment and confirm the previous work with studies using expression systems [11]. Because of the emerging role for Mct1 vesicles as a key component of the regulatory mechanism that controls the transporter's function, such work is needed to characterize them and their role in the regulation of Mct1 by cAMP.

To this end, the objectives of the present study were 1) To design and verify the utility of a fluorescent protein tagged Mct1 fusion protein as a valid marker for examining the vesicular dynamics of Mct1 in living RBE4 cells; 2) To characterize basic aspects of Mct1 vesicles and their dependence on the intracellular termini of the transporter; and 3) To evaluate cAMP dependent changes in the vesicular trafficking of the Mct1 fusion protein.

## Materials and Methods

### Cell culture

RBE4 cells, a gift of F. Roux [12], were cultured as described previously [6], [7], [8]. Briefly, cells were grown on collagen-coated polystyrene tissue culture dishes or collagen-coated number 1 coverslips in minimum essential medium alpha and Ham's F-10 nutrient (1∶1) with 10% fetal bovine serum, 1% antibiotic–antimycotic, 0.3 mg/ml geneticin, and 1.0 ng/ml basic fibroblast growth factor. Cells were trypsinized, replated between 3 and 8 hours prior to experimentation and used at subconfluency.

### Cytochemistry

To visualize fusion protein expression in fixed RBE4 cells, cells were transfected with expression vectors as described below and fixed at room temperature in 3.7% formaldehyde/phosphate buffered saline (PBS), rinsed and mounted. In dual transfection-immunodetection experiments, transfected fixed cells were rinsed with PBS, permeabilized with 0.5% TritonX-100 for 2.5 minutes at room temperature, blocked with 1.5% goat serum and stained overnight at 4°C with an anti-Rab5 antibody (diluted 1∶1000, Cell Signaling) or an anti-syntaxin-6 antibody (diluted 1∶1000, Cell Signaling). Primary antibodies were detected with Alexafluor-488 conjugated secondary antibodies (diluted 1∶125, Molecular Probes). For immunodetection of Mct1, cells were rinsed in PBS, permeabilized at room temperature as described in the text, blocked with 1.5% goat serum, and stained overnight at 4°C with an anti-Mct1 antibody (diluted 1∶1000, Millipore MP1286). The Mct1 primary antibody was detected with Alexafluor-488 conjugated secondary antibody (diluted 1∶125, Molecular Probes). All slides were mounted in ProLong Gold Antifade Reagent (Molecular Probes).

### Confocal and epifluorescence microscopy

Confocal images were taken on a Leica SPE or an Olympus FV1000 confocal microscope using 63 or 60X oil immersion objectives, respectively. The confocal aperture was set at 1 Airy Unit to optimize the confocality of the images and single planes from a basal region of the cells were acquired. Fluorophores were excited with the appropriate lasers, 488 or 561 or 543 nm, and fluorescence emission was filtered with the appropriate band pass filters (FV1000) or an acousto-optical tunable filter (SPE), and detected with a CCD camera (FV1000) or high sensitivity spectral detector (SPE) using the manufacturer's acquisition software. Epifluorescence video images were acquired with an Olympus IX50 microscope fitted with a 100 watt mercury arc lamp, a 100X oil immersion objective, appropriate narrow band pass filters, a Hamamatsu digital cooled IEEE139 CCD camera, and Imaging Workbench Ver. 5.2 Software (INDEC Systems, Inc.).

### Intracellular pH imaging

Ratiometric intracellular pH Imaging was conducted as described previously [6], [7], [8]. Cells were cultured on glass coverslips and loaded in HEPES-Buffered Saline (HBS) with 1 µM BCECF-AM for 20 minutes, and rinsed with fresh HBS before imaging with the Olympus IX50 microscope described above.

### Determination of the area, relative pH, and velocities of Mct1 vesicles

RBE4 cells were transfected with mCherry-Mct1 plasmids as described below and loaded with BCECF-AM as described above. Live cells were imaged using the Leica SPE confocal microscope. Eight-bit images for each color channel were acquired and mCherry-Mct1 images were digitally thresholded and processed using the Integrated Morphometry Analysis function of MetaMorph Image Analysis software version 7.7.4.0 (Molecular Devices) to produce regions of interest (ROI's) that corresponded to individual Mct1 vesicles (Figure S1). The area of the vesicles was calculated by the software. For pH measurements, the ROI's were superimposed on the corresponding raw BCECF images and the intensity of the average BCECF fluorescence was measured for each ROI (f_vesicle_). A single additional region of interest was hand drawn around the perimeter of each cell and the average BCECF fluorescence was measured (f_cell_). Each f_vesicle_ was divided by the corresponding f_cell_ to produce a ratio representing the pH of the vesicle relative to the pH of the entire cell. Our previous intracellular pH imaging work has shown the pH represented by f_cell_ to be 7.42+/−0.02 [7]. To quantify the velocities of Mct1 vesicles, epifluorescence video micrographs like that shown in supplemental video 1 (Video S1) were analyzed using the Track Objects function of the MetaMorph Image Analysis software.

### Molecular cloning and transfection

The rat Mct1 nucleotide coding sequence was RT-PCR amplified from RBE4 cell total RNA using the RETROScript reverse transcription kit (Ambion). PCR primers (polylinkers) containing EcoR1 (forward) and Sal1 (reverse) restriction sites were designed to amplify the full length Mct1 coding sequence from cDNA as follows: forward - 5′GCCGAATTCAAT GCCACCTGCGATTGGC; reverse - 5′CCCGTCGACTCAGACTGGGCTCTCCTC. The Mct1 C-terminus deletion PCR product was similarly amplified with the same forward primer and a reverse primer containing a Sal1 restriction site: 5′CCCGTCGACTCGATAATTGATGC CCAT. Mct1 PCR products were subcloned into the pmCherry-C1 vector (Clontech) using EcoR1 and Sal1 and the completed expression vectors were sequenced by SeqWright (Houston, TX). The Mct1 N-terminus deletion sequence, in a synthetic MiniGene (Integrated DNA Technologies), was subcloned into the full-length mCherry-Mct1 plasmid using EcoR1 and PpuM1. The dual tagged EGFP-mCherry-Mct1 construct was a modification of the full-length mCherry-Mct1vector produced by subcloning EGFP sequences that were PCR amplified from the pEGFP-C1 vector (Clontech) using Sac1 and EcoR1 polylinkers. Plasmids were transfected into RBE4 cells using the Qiagen Attractene Transfection Reagent and the manufacturer's protocol.

### Reagents

All reagents, unless otherwise specified, were purchased from Sigma Aldrich (St. Louis).

## Results

### Design and verification of a Mct1 fusion protein as a marker of Mct1 vesicular dynamics

**Figure 1:**
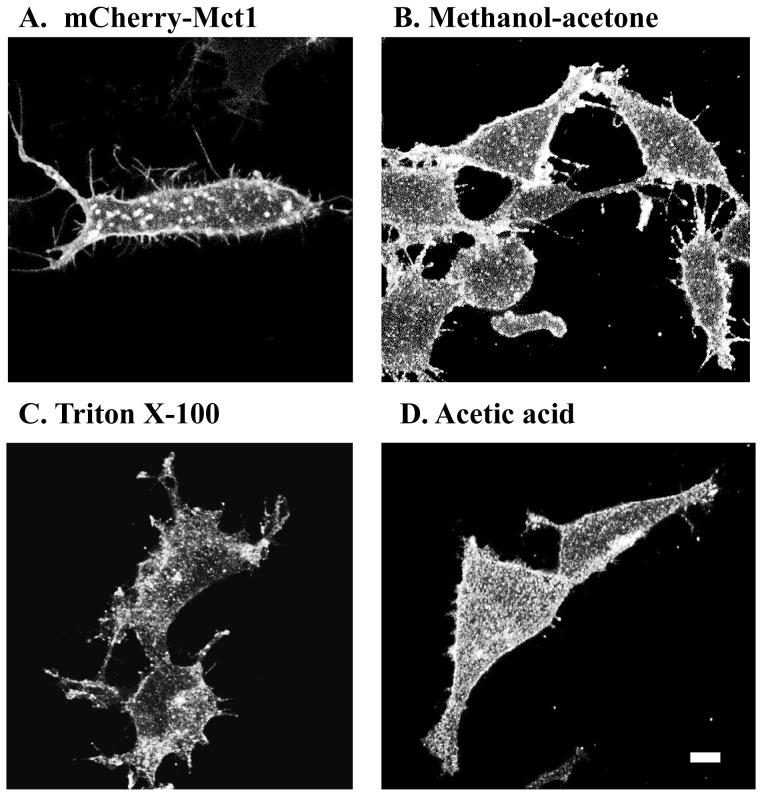
Mct1 is apparent in cytoplasmic vesicles. RBE4 cells expressing mCherry-Mct1 were imaged live using confocal microscopy (**A**). In the last three micrographs, RBE4 cells were immunostained with an anti-Mct1 antibody, fixed in 3.7% formaldehyde, and permeabilized with either methanol and acetone (**B**), or 0.5% TritonX-100 (**C**), or 5% glacial acetic acid (**D**). Scale = 5 µM.

A mCherry-Mct1 expression vector was designed to produce a protein product that would link mCherry to the N terminus of Mct1 with no other modifications to the transporter. The full length rat Mct1 DNA sequence was RT-PCR amplified from RBE4 cells, subcloned into the pmCherry-C1 expression vector (Clontech) and sequenced. The expression product was then tested for its distribution, function, and expression level in transiently transfected RBE4 cells. Examination of individual planes from confocal micrographs of living RBE4 cells clearly showed mCherry-Mct1 in numerous prominent puncta of various sizes and shapes, on the plasma membrane of basal, mid, and apical levels, and in lamellipodia and filopodia of the cells (Figure 1A). To examine whether the expression construct produced a pattern of Mct1 expression consistent with the native pattern, micrographs from transfected cells were compared with those from non-transfected cells that were fixed and immunostained for Mct1 using previously published methods and variations in the fixation procedure. The pattern of staining was comparable with that in immunostained cells, however, the puncta in the immuostained cells showed subtle changes in morphology among samples fixed by different methods (Figure 1 B-D). This verified that the appearance of Mct1 in cytoplasmic puncta was not simply an artifact of the methodology used to visualize it and showed a native expression pattern for the fusion protein.

**Figure 2:**
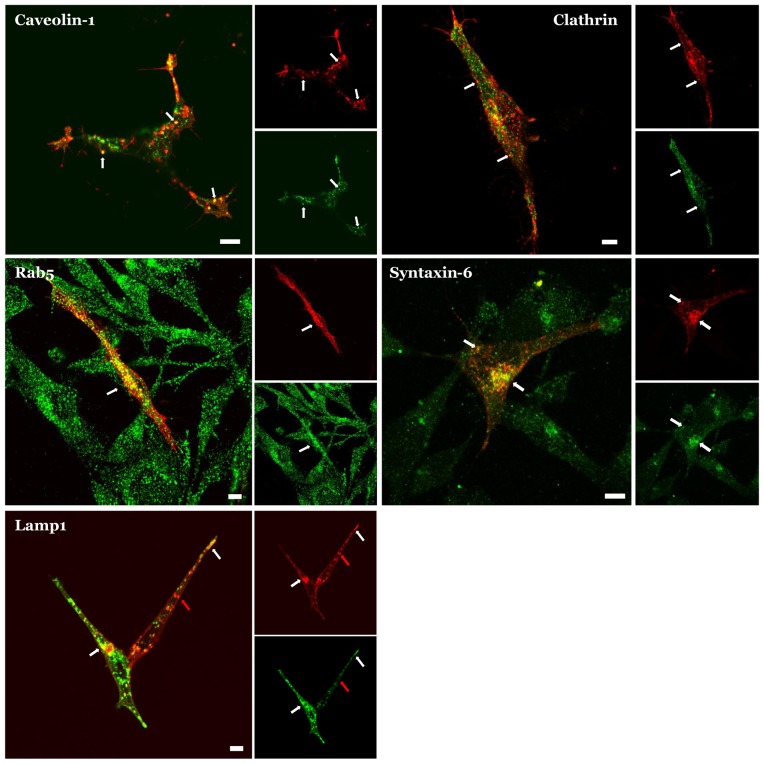
Colocalization of mCherry-Mct1 with endosomal markers. Each three part panel shows a large merged image and smaller images of the corresponding color channels, where Mct1 is pseudocolored red, the counter stain appears green, and colocalization appears yellow. Examples of colocalization are indicated by white arrows throughout. A single confocal plane near the basal region of the cells is shown in each case. mCherry-Mct1 co-localized with: YFP-caveolin-1 in puncta that were present near the plasma membrane and in the cytoplasm (upper left); GFP-clathrin in puncta near the plasma membrane and in cytoplasmic puncta (upper right); an anti-Rab5 antibody in numerous puncta (middle left); an anti-syntaxin-6 antibody in a cluster consistent with the trans-golgi and in cytoplasmic puncta (middle right); and GFP-Lamp1 in cytoplasmic puncta (lower left). Mct1 was also present in puncta that did not co-localize with Lamp1 (lower left, red arrow). Scale bars = 5 µM.

To further evaluate the fusion protein's pattern of expression we co-expressed it in RBE4 cells with markers for specific types of endosomes and imaged it in live cells. In these experiments, mCherry-Mct1 colocalized in puncta with GFP tagged clathrin, YFP tagged caveolin-1, and GFP tagged LAMP-1, consistent with previous results using immunocytochemistry [8]. We also found a similar result in mCherry-Mct1 expressing cells which were fixed and counter immunostained with antibodies to Rab5, or syntaxin-6 (Figure 2). The colocalization of mCherry-Mct1 with each of these markers confirmed the presence of Mct1 in different types of cytoplasmic vesicles as previously reported.

Next, to test whether the fusion protein was overexpressed, which can cause artifacts when examining vesicular trafficking, we expressed and visualized mCherry-Mct1 in live cells after titration of the expression vector over a range of concentrations. The expression pattern was not changed at titers higher than those used in the following studies, or when lowered to the detection limits of confocal microscopy. This result supported the conclusion that the fusion protein was not overexpressed (Figure S2).

To further evaluate this, we then expressed the fusion protein and measured its functionality in RBE4 cells using BCECF-AM imaging, which reports Mct1 transport function as an initial rate of cytoplasmic acidification in response to the application of extracellular lactate [6], [7], [13]. As an internal control, cells that expressed mCherry-Mct1 were paired within single fields of view with cells that expressed only native Mct1 and the measurement was compared among cells across a series of experiments. Demonstrating the functionality of mCherry-Mct1 as a lactic acid transporter, the initial rate of cytoplasmic acidification was increased by 13% in cells that transiently expressed the fusion protein (p = 0.002 in a paired t-test, N = 3 experiments, 10 cells per group).

Combined, the bright fluorescence of mCherry-Mct1, the similarity between its expression pattern in immunostains, its colocalization with markers of various types of endosomes, the stability of the fusion protein's expression pattern at a very low plasmid titration, the confirmation of its transport functionality, and the low level gain of function in cells that expressed the fusion protein, strongly suggested that mCherry-Mct1 was not overexpressed, trafficked similar to native Mct1, and therefore could be used as a useful marker to follow the vesicular trafficking of Mct1 in RBE4 cells.

### Characteristics of Mct1 vesicles and their dependence on the intracellular termini of Mct1

**Figure 3:**
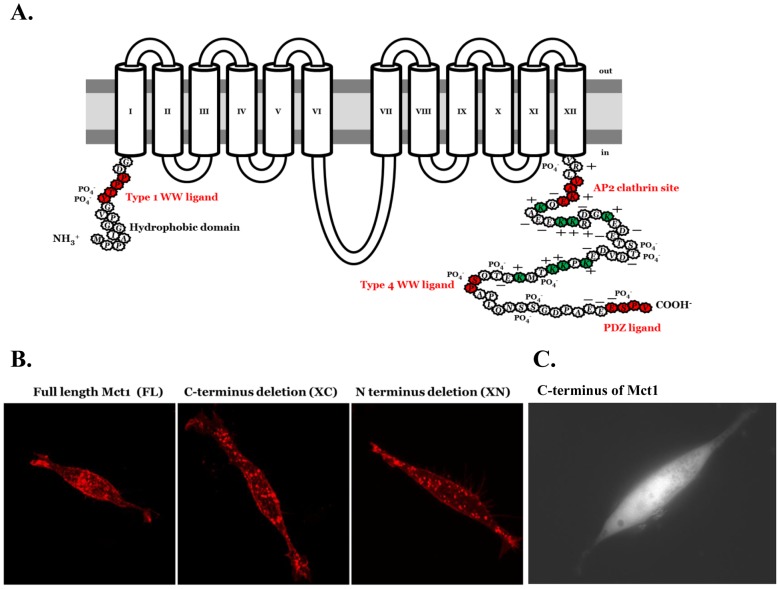
Characterization of Mct1 expression patterns in cells transfected with full length and deletion mCherry-Mct1 expression constructs. **A.** Protein motifs in the C and N termini of Mct1 that could be involved in controlling its localization to vesicles include (in red); type 1 and 4 WW ligands, an AP2 clathrin interaction site, a PDZ ligand, a hydrophobic N terminus, a charged C terminus (+ and −), lysine residues (shown in green), and numerous phosphorylation sites (PO_4_^−^). **B.** Confocal micrographs showed a similar appearance of Mct1 vesicles among cells expressing FL, XC, and XN mCherry-Mct1. **C.** An epi-fluorescence micrograph of an RBE4 cell expressing the C-terminus of Mct1 with mCherry fused to its amino terminus.

Because the peptide sequence of Mct1 has a number of elements in the intracellular N and C-terminal domains that could facilitate its localization to vesicles (Figure 3A), we deleted the N terminus (XN) or C terminus (XC) from the full-length (FL) mCherry-Mct1 fusion construct and transiently expressed each in RBE4 cells to see whether the deletion constructs would traffic normally. The basic mCherry-Mct1 staining pattern was not obviously changed by deletion of either terminus when examined in confocal micrographs (Figure 3B). Moreover, when the C-terminal sequence of Mct1 was isolated and expressed as a mCherry fusion protein the fluorescent product did not localize to vesicles or the plasma membrane, but instead was expressed diffusely in the cytosol and excluded from punctate regions (Figure 3C). A similar construct with a scrambled C terminal amino acid sequence appeared the same (not shown). Thus, at first glance the cytoplasmic termini did not appear to be important for localizing Mct1 to cytoplasmic vesicles.

Epifluorescence video micrographs of RBE4 cells transiently expressing mCherry-Mct1 showed the Mct1 vesicles to be highly dynamic, heterogeneous in size and velocity, and interactive with one another and the plasma membrane (Video S1). In the videos, two groups of vesicles were apparent upon inspection; a small-fast, and a large-slow moving population. To characterize this, video images from several cells were intensity-thresholded to show regions of interest (ROI's) corresponding to clearly identifiable individual vesicles, the area of each ROI was calculated, the regions were labeled (Figure S1 and materials and methods), and the vesicles divided into a small and a large half (0.82+/−0.04 and 2.72+/−0.2 µm^2^, N = 51 vesicles per group, p<0.001). Object tracking analysis showed that the smaller vesicles moved nearly twice as fast as the larger vesicles (0.37+/−0.03 and 0.72+/−0.05 µm/s, p<0.001). Thus, two populations of Mct1 vesicles were discernible by size and velocity. Vesicles increase in size during trafficking through the endosome/lysosome system [14]. Consistent with this, measurements of the areas of Rab5 and Lamp-1 puncta in micrographs of RBE4 cells showed the ratio of the Lamp1/Rab5 areas to be 1.8 (n = 5 cells each with 472 Lamp-1 and 1,361 Rab5 positive vesicles p = 0.01). Therefore, the larger slower vesicles likely more closely resemble lysosomes and the smaller faster vesicles likely represent early endosomes or close intermediates in the endosome/lysosome system.

**Figure 4:**
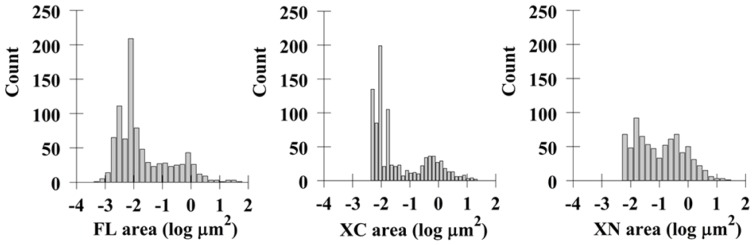
Histograms of the areas of Mct1 vesicles in RBE4 cells expressing FL, XC, and XN mCherry-Mct1. Deletion of the termini caused a rightward shift in the size distribution for the XC and XN groups with disappearance of the smallest vesicles. Average vesicular sizes for each group were FL = 0.47+/−0.1, XC = 0.53+/−0.06, XN = 0.57+/−0.06 µm^2^ (means and standard errors are given with n = 880 FL, 924 XC, and 759 XN vesicles).

To characterize the possible role of the intracellular termini in determining the size of Mct1 vesicles, images from single confocal planes taken just above the base of the cell were thresholded and ROI's were generated for analysis of their area as described above. One way analysis of variance (ANOVA) showed the areas of the XC-mCherry-Mct1 and XN-mCherry-Mct1 ROI's to be statistically significantly larger than those generated with FL-mCherry-Mct1 (p<0.001). Histograms of the size distributions showed that deletion of the intracellular termini caused the smallest vesicles to disappear (Figure 4). Thus, the termini of Mct1 may have importance for determining the more subtle aspect of the transporters localization to different sized vesicles.

**Figure 5:**
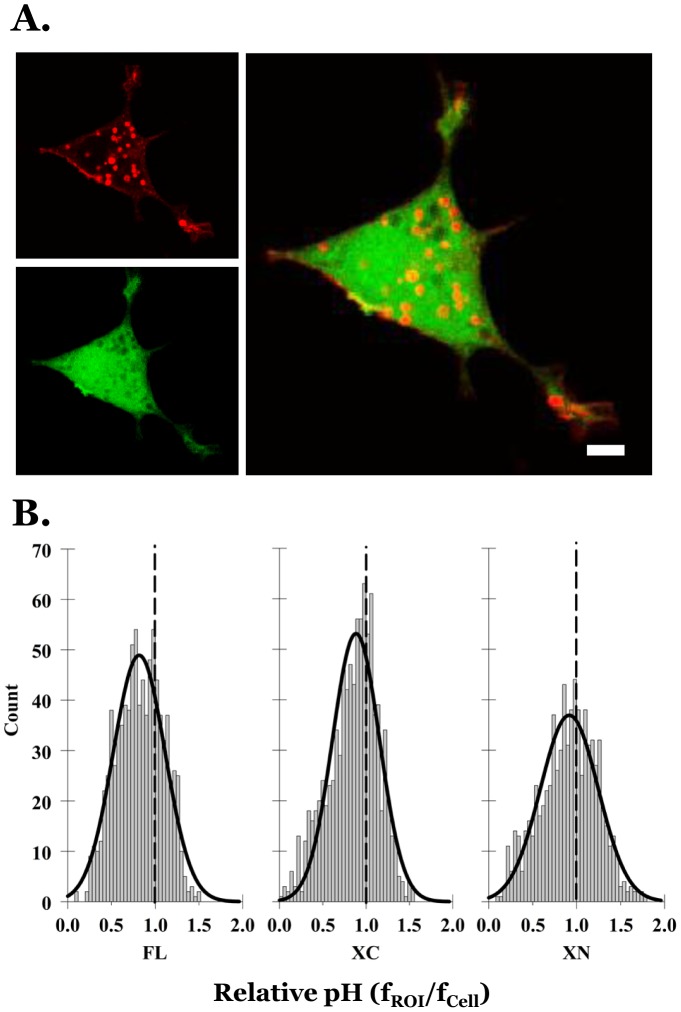
Mct1 vesicles spanned a pH range that was acid shifted relative to the overall pH of the cell. **A.** A single confocal plane from a RBE4 cell showing FL mCherry-Mct1 fluorescence (upper left) and the same plane showing BCECF fluorescence (lower left). As illustrated in the merged image (right), voids in BCECF staining often, but not always, colocalized with Mct1 vesicles. **B.** Histograms showing the distributions of the relative pH of Mct1 vesicles from 10 cells in each group expressing FL mCherry-Mct1 (880 vesicles), XC mCherry-Mct1 (924 vesicles), and XN mCherry-Mct1 (759 vesicles). Each distribution was fit with the Gaussian equation described in the text and is shown here as curves. Dashed lines show the average pH of the cells with relatively alkaline vesicles to their right and relatively acidic vesicles to the left. The mean relative pH's and standard errors were FL = 0.83+/−0.01, XC = 0.85+/−0.01, and XN = 0.91+/−0.01. Scale bars = 5 µM.

In a second approach aimed at exploring whether the termini of Mct1 might control sorting to different types of vesicles, we transiently transfected RBE4 cells with the XC, XN, or FL-mCherry-Mct1 constructs and counter stained with the intracellular pH indicator BCECF-AM. BCECF fluorescence was uneven in living RBE4 cells imaged at 100X, showing brighter staining in the nuclear region and numerous dark and bright puncta in the cytoplasm (Figure S3). Many, but not all of the mCherry-Mct1 vesicles perfectly co-localized with the dark puncta (Figure 5A). Therefore, we evaluated the confocal micrographs for colocalization of mCherry-Mct1 with the level of BCECF fluorescence. This was done by superimposing vesicular ROI's, generated with mCherry-Mct1, on images from the same planes visualized with BCECF. The average BCECF fluorescence intensity for each ROI (f_vesicle_) was then divided by the average BCECF intensity of the entire cell (f_cell_) to give an idea of its relative pH (see also materials and methods). Histograms of the relative pH of the vesicles were normally distributed around f_cell_ with slightly acid-shifted peaks (Figure 5B). For comparison, each distribution was fit with the Gaussian equation 
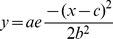
and shown as curves in the figure. Deletion of the termini of Mct1 caused the distributions to move in an alkaline direction with the effect being most dramatic in the XN group. ANOVA showed the mean relative pH values among the three groups to be statistically significantly different from one another (p<0.001). Therefore, the intracellular termini of Mct1 appeared to play a role in determining the pH distribution of vesicles to which the transporter localizes.

Overall, the above experimentation showed the Mct1 vesicles to span a range of sizes, velocities, and pH values, and the distributions over these parameters depended on elements in the C and N terminus of the transporter.

### Characterization of the effect of intracellular cAMP on Mct1 vesicles

**Figure 6:**
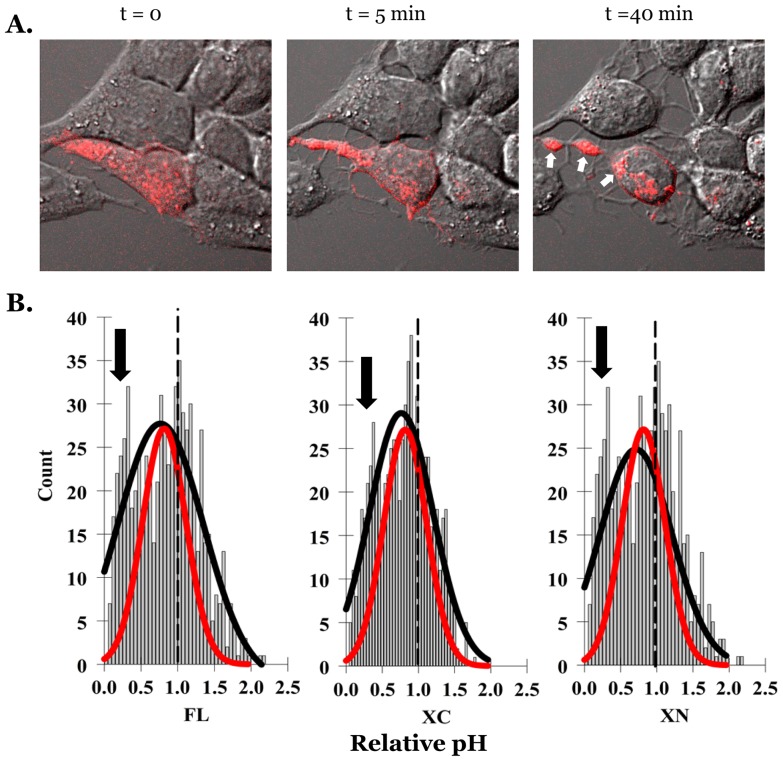
cAMP dependent changes in the localization and relative pH of mCherry-Mct1 vesicles in RBE4 cells. **A.** Excerpts from a video experiment with DIC images of RBE4 cells superimposed upon single confocal planes showing mCherry-Mct1 vesicles (red). The cells were exposed to 500 µM 8Br-cAMP at the start of a video imaging experiment, t = 0 min, and changed their morphology and distribution of Mct1 vesicles by the end of the experiment. Arrows show the location of three clusters of Mct1 vesicles that formed and became stationary during the cell's response. **B.** Histograms showing the distribution of the relative pH of Mct1 vesicles in RBE4 cells expressing FL mCherry-Mct1 (418 vesicles), XC mCherry-Mct1 (454 vesicles), and XN mCherry-Mct1 (589 vesicles). The histograms were fit by the Gaussian equation described in the text (black curves). As a reference, curves generated from the untreated control cells in figure 5 were scaled and superimposed on the graph (red curves). Arrows show the approximate positions of acidic peaks which appeared with cAMP treatment.

To investigate the potential effect of cAMP on the vesicular trafficking of Mct1, we treated FL-mCherry-Mct1 expressing RBE4 cells with a membrane permeant cAMP analog and videotaped mCherry fluorescence in confocal planes near the base of the cells over a 50 minute period beginning with initial treatment. Figure 6A and supplemental video 2 (Video S2) show a typical RBE4 cell responding by rapidly detaching from neighboring cells, rounding up, and forming prominent clusters of Mct1 vesicles within large processes that developed and extended from the main body of the cell. During the response, the clustered vesicles appeared to become nearly stationary while a group of smaller vesicles in the center of the cell remained mobile. Throughout the response, Mct1 was clearly visible on the plasma membrane and was prominent in numerous filopodia. Thus, cAMP caused changes in RBE4 cell morphology and clustering of Mct1 vesicles.

We then repeated the experiment in figure 5 with the addition of an experimental group that was treated with the cAMP analog to evaluate cAMP dependent changes in the relative pH of the population of Mct1 vesicles. Treatment caused a marked broadening of the relative pH distributions, with a slight overall shift to more acidic values (Figure 6B). This shift was not due to a cAMP dependent change in the cytosolic fluorescence (f_cell_) since this parameter decreased slightly with cAMP and therefore would have contributed to underestimating the acid shift (data not shown). In unpaired t-tests, the mean relative pH values were statistically significantly shifted in an acidic direction by cAMP within the XC and XN groups from 0.88 to 0.79 (p<0.001), and 0.94 to 0.77 (p<0.001), respectively. Interestingly, the mean relative pH of vesicles visualized with FL mCherry-Mct1 did not shift appreciably with cAMP, moving only from 0.85 to 0.84 (p = 0.56), however, broadening of the range was most pronounced in this group. In each case the cAMP dependent change in the pH distribution appeared to include emergence of an acidic peak (indicated by arrows in Figure 6B). This result is consistent with cAMP changing the intracellular trafficking of Mct1, with some vesicles moving to more acidic compartments by a mechanism involving elements in the intracellular termini of the transporter.

**Figure 7:**
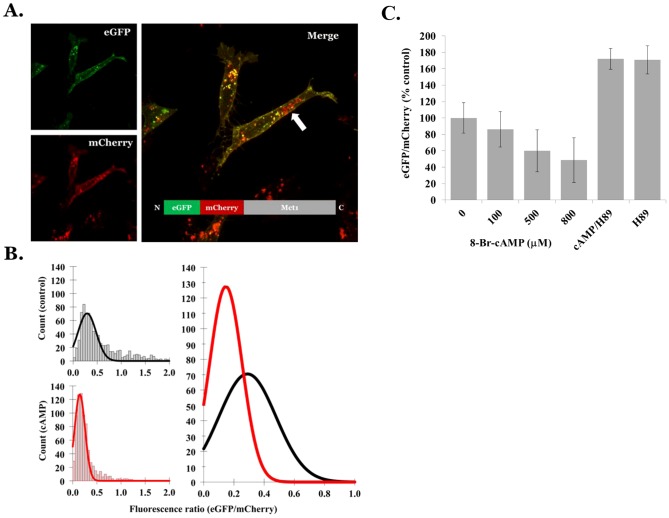
cAMP dependent acidification of dual tagged Mct1 vesicles. **A.** A confocal micrograph of EGFP-mCherry-Mct1, showing individual EGFP (green) and mCherry (red) color channels from a single plane on the left and the merged image on the right. The inset on the right diagrams the protein product of the expression construct. The arrow indicates a cluster of Mct1 vesicles that appear red because of selective quenching of the EGFP signal in more acidic endosomes. **B.** Histograms showing the distributions of the EGFP/mCherry intensity ratios in vesicles from control (n = 7 cells, 828 vesicles, upper left) and 500 µM 8Br-cAMP treated cells (7 cells, 748 vesicles, lower left) and curves fit with the Gaussian equation described in the text. The curves are shown superimposed in the right panel for comparison of results between control (black) and 8Br-cAMP treated cells (red). **C.** Dose response showing the effect of 8Br-cAMP on control-normalized EGFP/mCherry intensity ratios measured 30 minutes after treatment. In the H89 groups, 20 µM H89 was present for 10 minutes with or without 500 µM 8Br-cAMP (n = 6 cells with >200 vesicles per group). The experiment was repeated with similar results.

Finally, to confirm the effect of cAMP on the pH of mCherry-Mct1 vesicles, we constructed a dual EGFP-mCherry tagged full length Mct1 expression construct, transiently expressed it in RBE4 cells, and evaluated acid dependent quenching of EGFP fluorescence as a ratio of the acid-insensitive mCherry fluorescence (green/red) [15], [16]. Because both fluorochromes were physically linked to Mct1 with 1∶1 stoichiometry, merged red-green images would be expected to produce a uniform yellow signal in regions of neutral pH and a predominantly red signal in more acidic regions. In transfected cells, EGFP-mCherry-Mct1 was present in cytoplasmic puncta, and on the plasma membrane as a yellow signal, however, a small group of red-only vesicles was also present in the cytoplasm, consistent with a population of more acidic endosomes which contained the transporter (Figure 7A). To verify the utility of the construct as a pH indicator, we then acidified the cytoplasm of RBE4 cells by incubating them in the proton ionophore nigericin at low pH/high K^+^ or in lactate. In these experiments, the green/red ratio was significantly diminished when the pH of the cytoplasm was lowered by either nigericin or lactate (Figure S4). This verified the utility of the construct as a pH indicator. Furthermore, verifying that the transporter sometimes localized to vesicles of lower pH, ratiometric images (green/red) showed large punctate voids in the cytoplasm which perfectly colocalized with the mCherry signal (Figure S4). Therefore, we proceeded to test the effect of treating RBE4 cells with the cAMP analog. After a 25 minute exposure, treated cells showed a decrease in the green/red ratio, consistent with an acid shift in the pH of the Mct1 vesicles (Figure 7B). The means of the ratios dropped dramatically and were statistically significantly different in one way ANOVA tests with control = 0.56+/−0.19 and cAMP = 0.26+/−0.05, p<0.001 (variances are given). The result was repeated in independent experiments showing a dose response for the cAMP analog. In addition, the presence of the selective PKA inhibitor, H89, had an alkalizing effect on Mct1 vesicles that was not affected by co-incubation with the cAMP analog. This confirmed a role for PKA in controlling the pH of Mct1 vesicles, as previously shown for the effect of cAMP in regulating Mct1 function (Figure 7C) [6]. Overall, these experiments confirmed our results with BCECF and supported the conclusion that cAMP causes Mct1 to traffic to acidic compartments.

## Discussion

### Basic characteristics of Mct1 vesicles

The major scientific objectives of this work were to provide a basic characterization of Mct1 vesicles in RBE4 brain microvascular endothelial cells, to elucidate how they are involved in the regulation of Mct1 function by cAMP, and to examine whether trafficking of Mct1 vesicles is dependent upon elements in the transporter's cytoplasmic N or C termini. This study confirmed that Mct1 vesicles are not byproducts of a particular immunostaining process, or an artifact of our fusion protein's expression. It also confirmed the heterogeneous nature of the Mct1 vesicles which colocalized with markers for caveolae, clathrin coated vesicles, early endosomes, trans-golgi, and lysosomes. Fluorescence video data showed the Mct1 vesicles to be highly mobile in the cell and identified a relationship between the size and velocity of the vesicles. The vesicles were also shown to span broad size and pH ranges as expected for transporters moving through the relatively alkaline secretory pathway to increasingly acidic compartments of various endosomal and degradation pathways [17], [18]. Combined, the results of these experiments showed the utility of our mCherry-Mct1 fusion protein for investigating the vesicular trafficking of Mct1, and pointed to a complex and heterogeneous population of cytoplasmic vesicles being involved in the cellular trafficking of Mct1 in RBE4 cells.

### Role for a degradation pathway in the cAMP dependent regulation of Mct1

Previous work in RBE4 cells demonstrated that β-adrenergic signaling through adenylyl cyclase, cAMP, and protein kinase A, rapidly decreases the level of Mct1 on the plasma membrane [6], [8]. This appears to involve caveolae, and ultimately decreases the transport function of Mct1 [8]. The study presented here extends our understanding of this regulatory pathway by showing that cAMP stimulates trafficking of Mct1, and increases the level of transporter found within more acidic endosomes. These results were consistent with regulatory pathways for other membrane proteins including β2-adrenergic receptors and glutamate transporters which are degraded in response to adrenergic signaling [19], [20]. We think the identity of the endosomes in our studies is most likely to be autophagosomes or lysosomes since the dual tag was fused to the endofacial surface of Mct1 and would be expected to face the cytoplasm as it traffics. Thus, only cytoplasmic acidification around a vesicle, or entry of the entire protein, or its vesicle, into an acidic compartment could have decreased the green/red fluorescence ratio in punctate patterns that we observed [21]. Since cAMP did not acidify the cytoplasm in our experiments, the latter interpretations seem most likely. This does not rule out the possibility of proteolytic removal and degradation of the fluorescent tag of our fusion proteins being stimulated by cAMP, however, this would still point to degradation of Mct1 being an endpoint of cAMP signaling in RBE4 cells. Thus, a more complete picture of the cAMP dependent regulation of Mct1 appears to include lysosomal degradation of the transporter as an endpoint in the regulatory process. Therefore, based on the previous literature, a more complete picture of the regulation of Mct1 involves cAMP stimulating its removal from the plasma membrane through a process involving its trafficking to caveolae, delivery to an endosomal trafficking pathway, and subsequent entry of a population of the transporters into autophagosomes or lysosomes where they would be degraded [6], [8].

### Role of its cytoplasmic termini in the vesicular trafficking and regulation of Mct1

An important aspect of this study was results implicating the intracellular termini of Mct1 in its vesicular trafficking and cAMP dependent regulation. In epithelial cells, Mct1 is targeted to particular membranes independent of particular sorting motifs in its intracellular regions, instead requiring CD147 as a chaperone to control its localization [22], [23], [24]. The reported irrelevance of endogenous signals in Mct1 for its trafficking is surprising given multiple putative protein-protein interaction and trafficking motifs in its intracellular termini (Figure 3A). Our result that the transporter remained in vesicles after deletion of its N or C termini, and that the isolated C-terminus did not localize to vesicles or the plasma membrane, was consistent with the studies in epithelium. However, our observation that deletion of either termini caused Mct1 vesicles to become larger and more alkaline provided evidence that the termini are involved in regulating Mct1 trafficking. Moreover, effects of cAMP were altered by deletion of the termini, pointing to a role for them in the cAMP dependent regulation of Mct1. These effects were most pronounced when the N-terminus was deleted, indicating that its hydrophobicity, previously demonstrated phosphorylation in brain at Y11 or T12 [25], or its type 1 WW ligand could be important determinants of Mct1 regulation by cAMP (Figure 3A). Future studies should be designed to evaluate this. It is also an interesting possibility that some of the effects on vesicular properties could be due to terminus-dependent changes in Mct1 functionality within the vesicular membrane, and this should be examined in future studies. To the best of our knowledge this report is the first to provide evidence of a trafficking or regulatory function for the intracellular termini of Mct1.

### Justification of the vesicular pH imaging assay developed in this study

While BCECF-AM is generally used as a cytoplasmic pH indicator, its use to measure the relative pH of cytoplasmic vesicles was a novel application developed in this study. This raised the question whether it truly reflected vesicular pH, or random variations in BCECF fluorescence in vesicles that failed to incorporate the dye. We think the method reported vesicular pH for the following reasons: 1) BCECF-AM has been previously shown to label alkaline organelles such as nuclei and mitochondria [26], [27], [28], [29]. Brighter staining of nuclei and fine puncta that looked like mitochondria were clearly observed, showing that the dye could access cytoplasmic organelles in our experiments (Figure 5A and Figure S3). 2) Some vesicles that were positive for mCherry-Mct1 showed bright BCECF fluorescence and so were clearly capable of incorporating the dye. 3) The heterogeneity in the relative pH of Mct1 vesicles and the shape of their pH distribution was strikingly similar to that of epithelial cell endosomes measured previously with FITC-dextran (Figure 5B) [27]. 4) cAMP acidified Mct1 vesicles when assessed by BCECF fluorescence and independently using our dual tagged Mct1 construct. Therefore, while our results do not rule out the possibility that some of the dark BCECF puncta simply excluded the dye, the most likely explanation for the cAMP dependent decrease in BCECF fluorescence of Mct1 vesicles would not be that treatment somehow improved their ability to exclude the dye, but rather led to their acidification which quenched the fluorescence.

### Importance of the vesicular trafficking of Mct1

Vesicular trafficking is well known to localize proteins to cellular microdomains where they are active, and so is an important mechanism controlling the function of membrane transporters, receptors, and channels [30], [31]. Vesicular trafficking is a particularly prominent feature in endothelial barriers where it provides protein sorting functions, facilitates trans-barrier movement of bulk fluids and solutes, and it is uniquely differentiated in brain to support cerebral homeostasis [32], [33]. However, despite its potential importance for understanding the normal physiology of Mct1 and in developing therapeutic targets for certain brain diseases, the regulation of Mct1 trafficking has largely gone unstudied in brain cells of any type. Cerebral microvascular endothelial cells *in vivo* are unique in many properties including the close juxtaposition of their luminal and abluminal membranes and high expression of tight junction proteins. Junctional proteins act as a scaffolding to localize various ion channels and transporters into microdomains, contributing to their regulation and overall cellular function, and so could be important for localizing Mct1 in brain microvessels [34]. While this will be important to investigate in future studies that more closely model the blood brain barrier *in vivo*, these structural attributes were not well modeled in our studies with isolated cultured RBE4 cells; rather, our work was designed to elucidate basic Mct1 regulatory and trafficking pathways occurring at a cellular level in the simplest cell system. Therefore, we believe the present study is more relevant as a model for the regulation of Mct1 during embryogenesis and in certain brain pathologies when endothelial cells become migratory; for example, during vascularization of damaged areas following stroke and brain injury, or during neovascularization of growing brain tumors [35], [36], [37]. Supporting this, Mct4, a close isoform of Mct1, was shown to associate with CD147 and β-integrin in a complex essential for epithelial cell migration, and inhibition of Mct1 blocked bovine aortic endothelial cell migration in a pathway involving hypoxia-inducible factor-1 signaling [37], [38]. Unpublished work from our lab has shown that the transport function of Mct1 and RBE4 cell migration are simultaneously blocked by either Mct1 inhibitors or agents that disrupt vesicular trafficking. This suggests regulation of Mct1 by a mechanism involving its vesicular trafficking may be critical for controlling brain endothelial cell migration, and therefore vascularization of brain tissue during development or pathology. As such, the elucidation of vesicular trafficking pathways that control Mct1 location and function in cultured brain microvascular endothelial cells presented here provides important information that could lead to specific targets for developing new therapeutic approaches for stroke, brain injury, and brain cancers, where changes in brain capillary cell migration and vascularization are key components of the etiological and healing processes.

## Supporting Information

Figure S1 **Example of the image processing used to make Mct1-vesicular ROI's.** On the left is a raw grey scaled 8 bit image from a single confocal plane in a basal region of an RBE4 cell that was transiently transfected with FL Mct1-mCherry. In the middle, the same data was filtered to show pixels above an intensity-threshold, 144 grey scale values in this case. Intensity-thresholds were chosen based on a point just above which pixels on the plasma membrane were excluded by the threshold. A threshold mask is shown in red, superimposed on the raw data, and can be seen to correspond to the portion of the raw image that shows mCherry-Mct1 vesicles. On the right, individually labeled ROI's, shown as green filled areas surrounded by a white border and superimposed on the raw image data, were generated from the threshold mask for analysis of the size and intensity of individual ROIs. Scale bar = 5 µM.
(TIF)

Figure S2 **The pattern of mCherry-Mct1 expression is stable over a range of plasmid concentrations.** On the left, the pixel intensity was averaged in maximum Z-projections from 8-bit confocal images within ROI's corresponding to individual cells and plotted against the percent plasmid used relative to that in all other transfections within this report. The result showed that the plasmid level used in this study (100%) produced a fluorescence level that on average was between +/−87% of that achieved with twice or half the amount of plasmid, respectively. It can also be seen that lowering the plasmid level below 100% produced images that on average rapidly approached detection limits. On the right are selected images of mCherry-Mct1 expressing cells transfected with various levels of the plasmid (indicated above each image). Membrane and vesicular staining can be clearly seen in each image regardless of the level of plasmid used.
(TIF)

Figure S3 **Example of BCECF fluorescence in bright puncta within the cytoplasm of RBE4 cells.** The cell was loaded with BCECF-AM as described in the materials and methods and imaged in a single basal plane using confocal microscopy. The nuclear region (right) and numerous puncta (left) can be seen to fluoresce more brightly than the surrounding cytoplasm. The smaller puncta are consistent in size and shape with mitochondria in RBE4 cells which were previously visualized with MitoTracker staining (not shown). Scale bars = 5 µM.
(TIF)

Figure S4 **The green/red ratio of the fluorescence from EGFP-mCherry-Mct1 expressing cells was a pH indicator.** Confocal stacks of individual RBE4 cells expressing EGFP-mCherry-Mct1 were acquired under standardized settings. Fluorescence intensities were summed across each stack and the green/red ratio was calculated. It can be seen that lowering the cytosolic pH by briefly incubating the cells in HEPES buffer with 15 µM Nigericin and 135 mM K^+^ substituted for Na^+^, or 20 mM L-lactate, caused a decrease in the green/red ratio (N = 10 or 11 cells/group, * p<0.05). The experiment was repeated twice with similar results (upper left). In background subtracted green/red ratiometric images, the lowest ratios appeared in puncta of cytoplasmic regions of the cells (dark regions on the right hand panels) and colocalized (green arrows) when superimposed on the mCherry channel from the same images (red, lower left).
(TIF)

Video S1 **Example of a RBE4 cell showing Mct1-mCherry expression on the plasma membrane and within mobile cytoplasmic vesicles.** Two populations of vesicles are apparent; a group of smaller faster moving vesicles that are localized near the center of the cell, and a group of larger slower moving vesicles that appear in the periphery. In the video, the large vesicles sometimes seem to interact and occasionally appear to exchange smaller vesicles with one another (inset). Vesicles are also present and mobile very close to the plasma membrane and sometimes appear interactive with it.
(MPG)

Video S2 **Dual phase contrast-confocal video microscopy showed the response to a cAMP analog of a RBE4 cell labeled with the full length Mct1-mCherry construct (red) over a 50 minute period.** As cells in a loosely associated island responded to 500 µM 8Br-cAMP, cytoplasmic vesicles appeared to coalesce and fuse with one another. In addition, the cells rounded up and retracted from contacts with their neighbors. During the retraction, Mct1 could be seen in filopodia that appeared to maintain connected with neighboring cells. Throughout the response, Mct1 expression on the plasma membrane and in cytoplasmic vesicles was maintained.
(MP4)
