# Supplementary figures and images for: Regulation of Monocarboxylic Acid Transporter-1 by cAMP Dependent Vesicular Trafficking in Brain Microvascular Endothelial Cells
Source: PLoS One. 2014 Jan 16;9(1):e85957. doi: 10.1371/journal.pone.0085957 (PMC3894203; doi:10.1371/journal.pone.0085957)

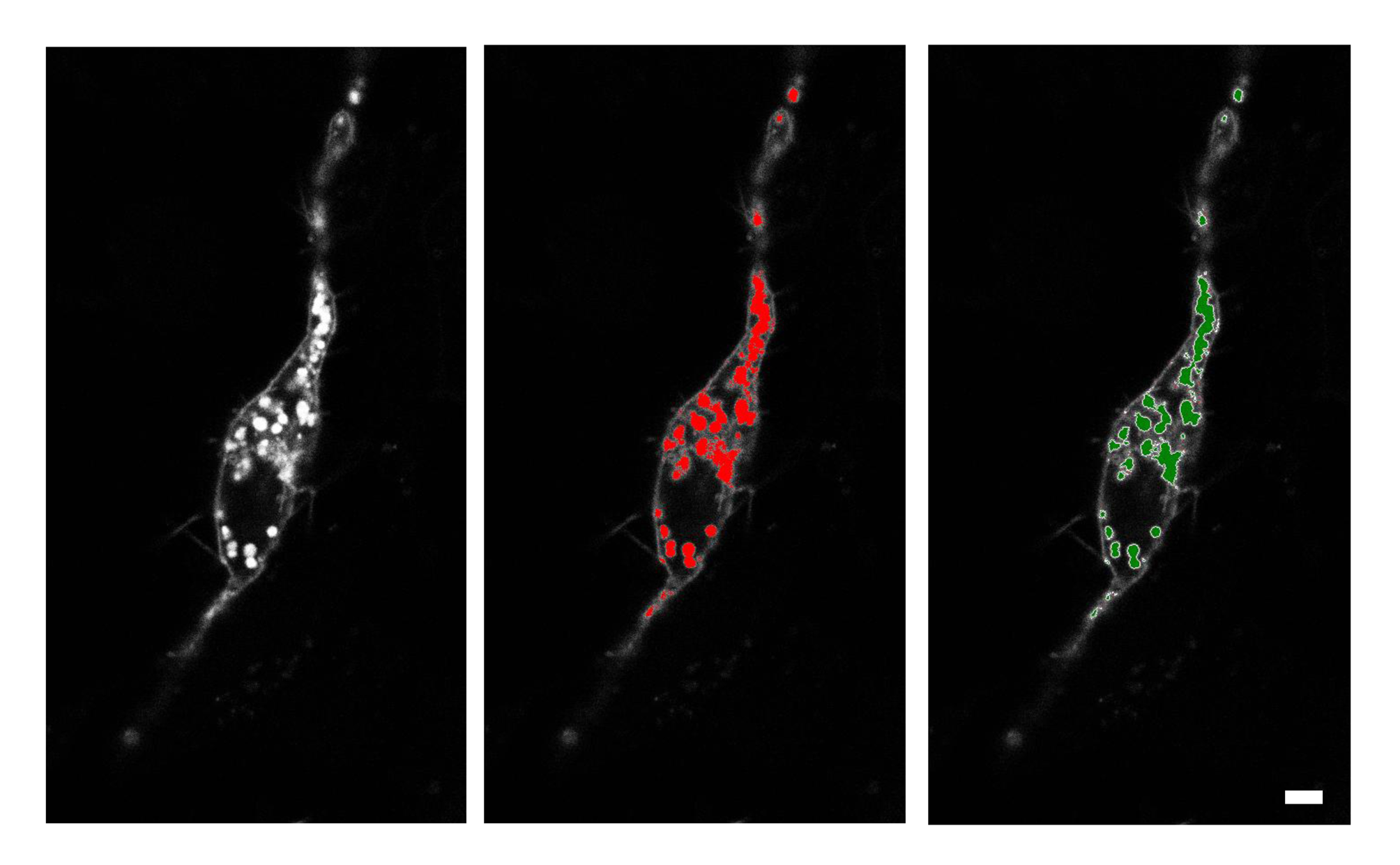

Supplement: Figure S1 — Example of the image processing used to make Mct1-vesicular ROI's. On the left is a raw grey scaled 8 bit image from a single confocal plane in a basal region of an RBE4 cell that was transiently transfected with FL Mct1-mCherry. In the middle, the same data was filtered to show pixels above an intensity-threshold, 144 grey scale values in this case. Intensity-thresholds were chosen based on a point just above which pixels on the plasma membrane were excluded by the threshold. A threshold mask is shown in red, superimposed on the raw data, and can be seen to correspond to the portion of the raw image that shows mCherry-Mct1 vesicles. On the right, individually labeled ROI's, shown as green filled areas surrounded by a white border and superimposed on the raw image data, were generated from the threshold mask for analysis of the size and intensity of individual ROIs. Scale bar = 5 µM. (TIF) [file pone.0085957.s001.tif]

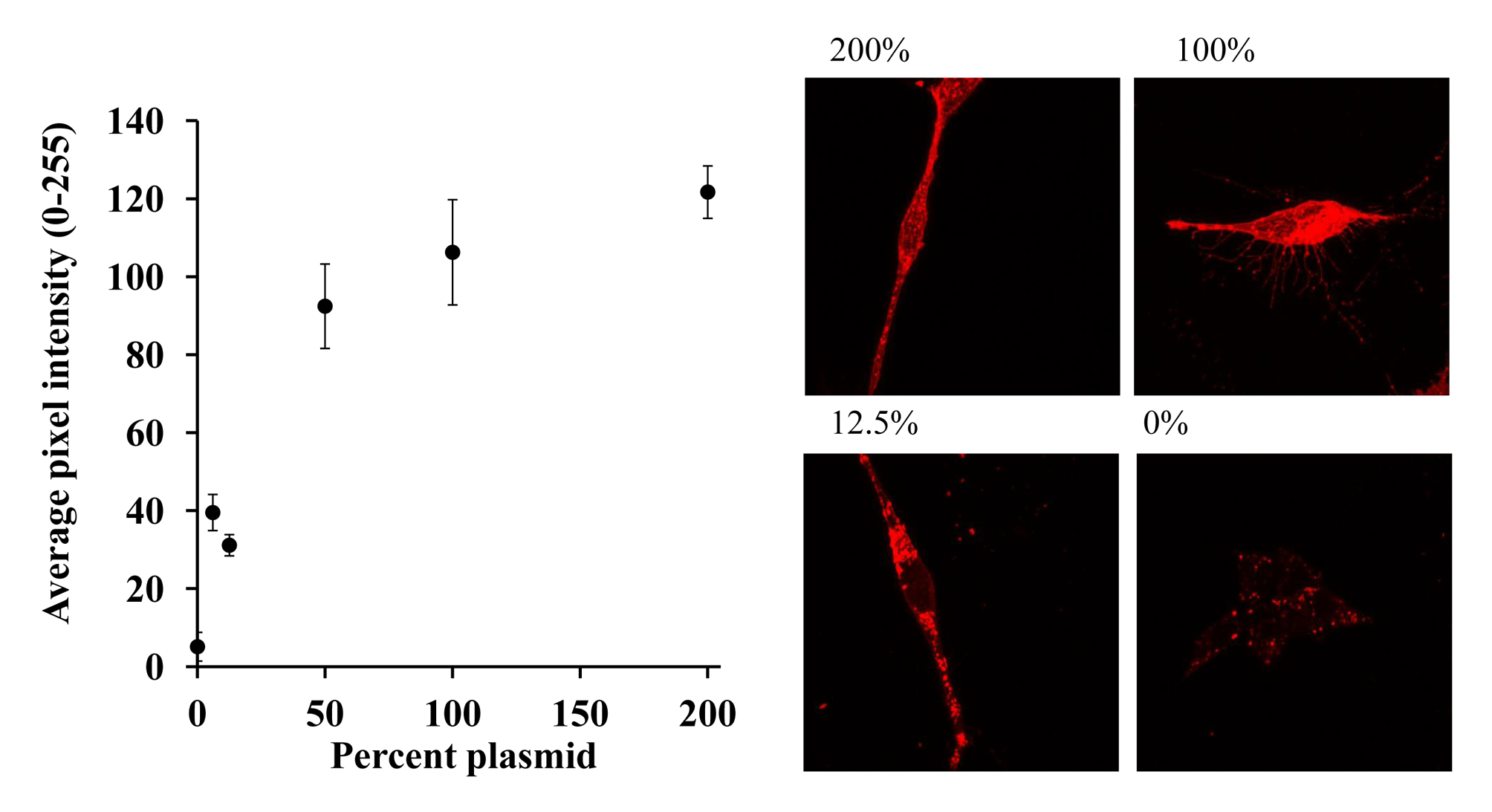

Supplement: Figure S2 — The pattern of mCherry-Mct1 expression is stable over a range of plasmid concentrations. On the left, the pixel intensity was averaged in maximum Z-projections from 8-bit confocal images within ROI's corresponding to individual cells and plotted against the percent plasmid used relative to that in all other transfections within this report. The result showed that the plasmid level used in this study (100%) produced a fluorescence level that on average was between +/−87% of that achieved with twice or half the amount of plasmid, respectively. It can also be seen that lowering the plasmid level below 100% produced images that on average rapidly approached detection limits. On the right are selected images of mCherry-Mct1 expressing cells transfected with various levels of the plasmid (indicated above each image). Membrane and vesicular staining can be clearly seen in each image regardless of the level of plasmid used. (TIF) [file pone.0085957.s002.tif]

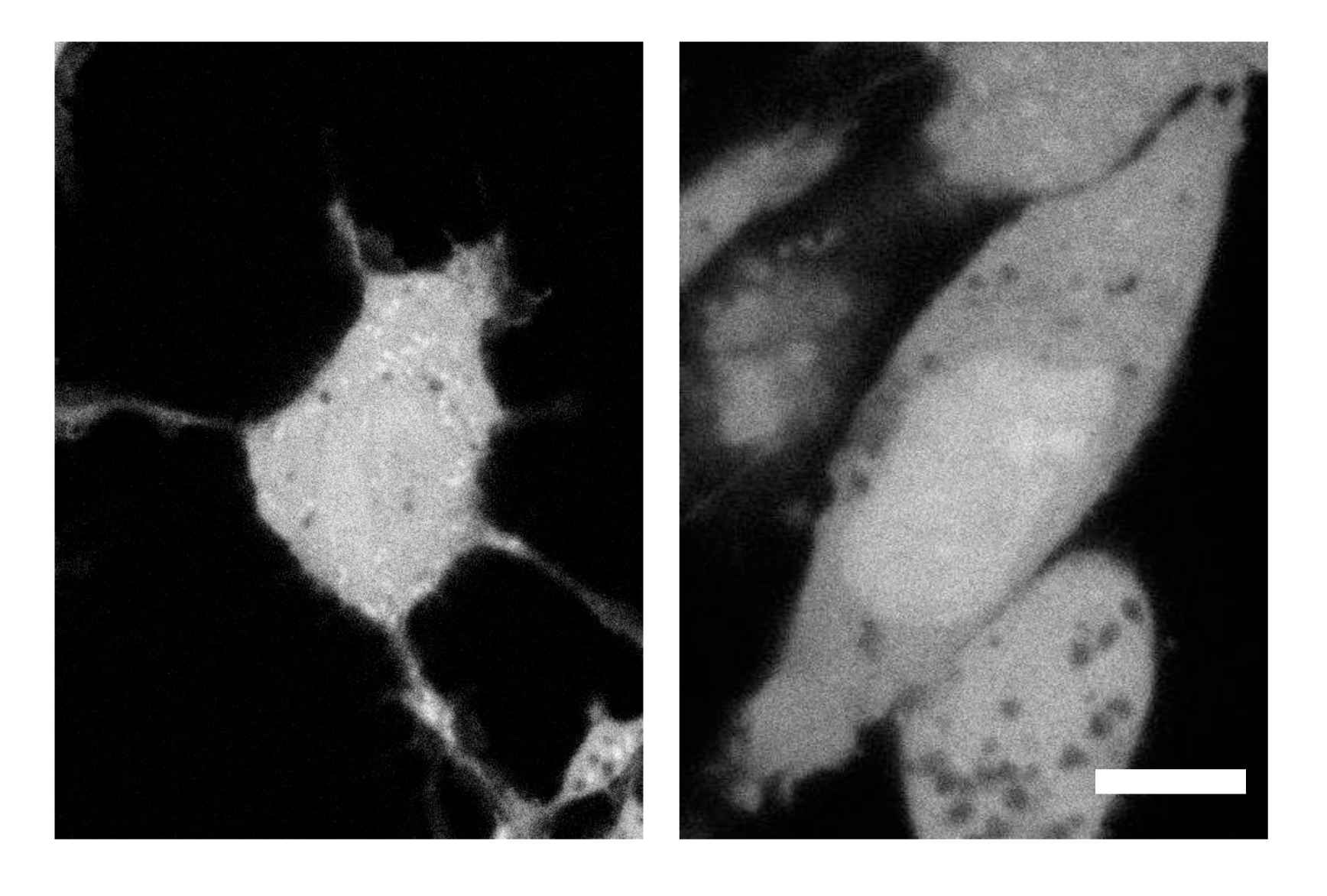

Supplement: Figure S3 — Example of BCECF fluorescence in bright puncta within the cytoplasm of RBE4 cells. The cell was loaded with BCECF-AM as described in the materials and methods and imaged in a single basal plane using confocal microscopy. The nuclear region (right) and numerous puncta (left) can be seen to fluoresce more brightly than the surrounding cytoplasm. The smaller puncta are consistent in size and shape with mitochondria in RBE4 cells which were previously visualized with MitoTracker staining (not shown). Scale bars = 5 µM. (TIF) [file pone.0085957.s003.tif]

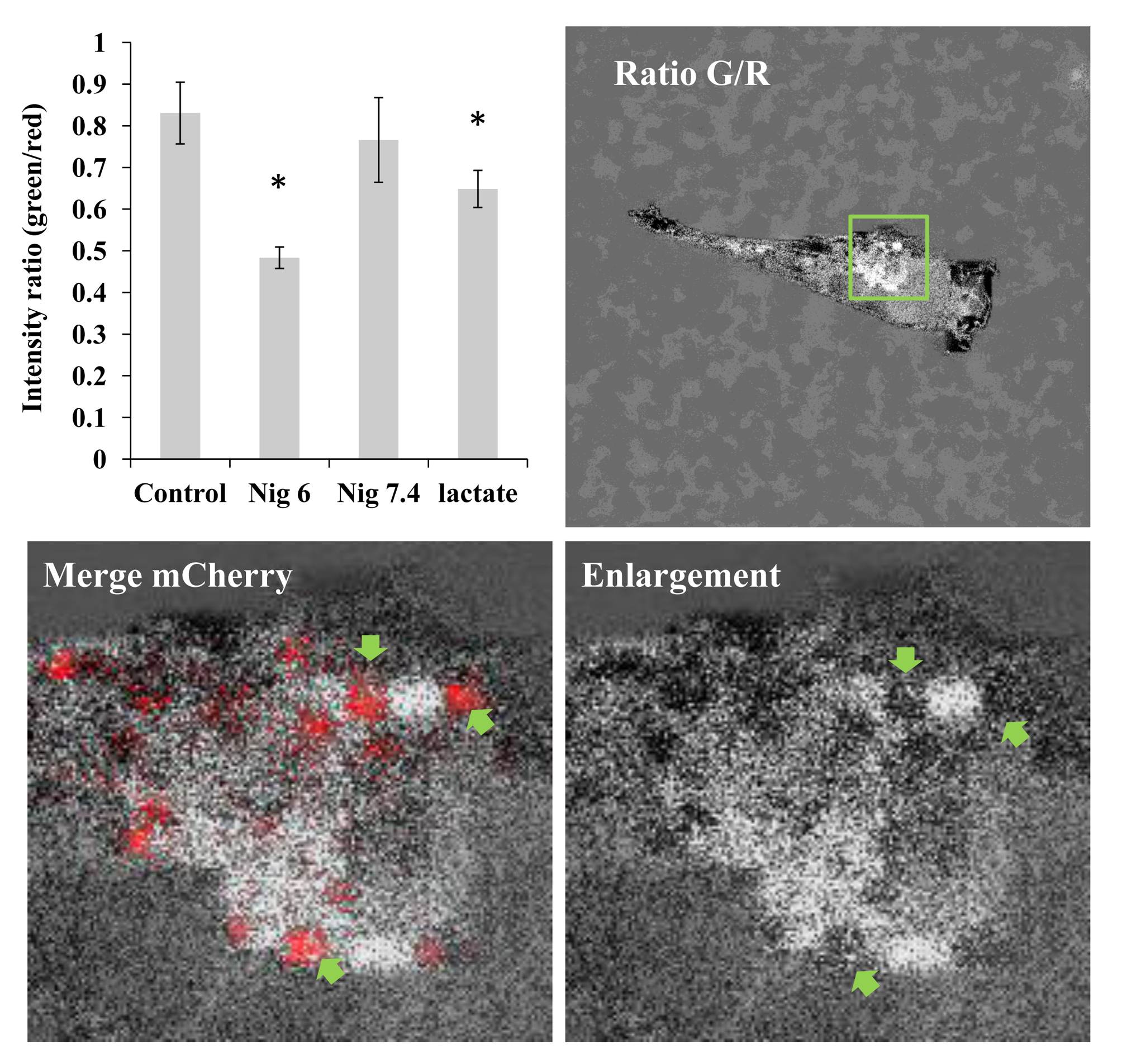

Supplement: Figure S4 — The green/red ratio of the fluorescence from EGFP-mCherry-Mct1 expressing cells was a pH indicator. Confocal stacks of individual RBE4 cells expressing EGFP-mCherry-Mct1 were acquired under standardized settings. Fluorescence intensities were summed across each stack and the green/red ratio was calculated. It can be seen that lowering the cytosolic pH by briefly incubating the cells in HEPES buffer with 15 µM Nigericin and 135 mM K+ substituted for Na+, or 20 mM L-lactate, caused a decrease in the green/red ratio (N = 10 or 11 cells/group, * p<0.05). The experiment was repeated twice with similar results (upper left). In background subtracted green/red ratiometric images, the lowest ratios appeared in puncta of cytoplasmic regions of the cells (dark regions on the right hand panels) and colocalized (green arrows) when superimposed on the mCherry channel from the same images (red, lower left). (TIF) [file pone.0085957.s004.tif]
